# A Strain-Based Load Identification Model for Beams in Building Structures

**DOI:** 10.3390/s130809909

**Published:** 2013-08-05

**Authors:** Kappyo Hong, Jihoon Lee, Se Woon Choi, Yousok Kim, Hyo Seon Park

**Affiliations:** Department of Architectural Engineering, Yonsei University, 50 Yonsei-ro, Seoul 120-749, Korea; E-Mails: kappyo@yonsei.ac.kr (K.H.); blithebard@naver.com (J.L.); watercloud@yonsei.ac.kr (S.W.C.); yskim1220@yonsei.ac.kr (Y.K.)

**Keywords:** strain sensing, load identification, structural health monitoring, steel beams

## Abstract

A strain-based load identification model for beam structures subjected to multiple loads is presented. The number of sensors for the load identification model is the same as the number of load conditions acting on a beam structure. In the model, the contribution of each load to the strains measured by strain sensors is defined. In this paper, the longitudinal strains measured from multiplexed fiber Bragg grating (FBG) strain sensors are used in the load identification. To avoid the dependency on the selection of locations for FBG sensors installed on a beam structure, the measured strain is expressed by a general form of a strain sensing model defined by superimposing the distribution shapes for strains from multiple loads. Numerical simulation is conducted to verify the model. Then, the load identification model is applied to monitoring of applied loads on a 4 m-long steel beam subjected to two concentrated loads. In the experiment, seven FBG sensors and nine electrical strain gages (ESGs) were installed on the surface of the bottom flange. The experimental results indicate a good agreement between estimated loadings from the model and the loads applied by a hydraulic jack.

## 1. Introduction

To secure the safety of a beam structure subjected to multiple loads such as gravity-induced loads, earthquakes, winds, or unexpected loads, the maximum stress in a beam structure is measured and checked not to ensure it does not exceed the allowable stress of a member [1,2]. That is the reason why strain-based structural health monitoring (SHM) has been widely used in sensing the maximum stresses of beam structures [3–6].

Using the measured strains from strain sensors, the safety of a beam structure can be managed by controlling the level of maximum strain. To control the level of strain in a beam structure, it is necessary to identify the magnitude of all the loads acting on the beam structure. Various techniques to estimate the magnitudes of loads acting on structures have been reported [7–10]. Unlike load identification methods based on modal tests for buildings or infra-structures, it is necessary to develop a practical identification method for loads acting on a beam structure in real operative conditions.

Long gauge fiber optic sensors (LGFOSs), vibrating wire strain sensors (VWSGs), and fiber Bragg grating (FBG) strain sensors have been utilized in the estimation of maximum strains of beam structures [4,11–15]. In the case of LGFOSs and VWSGs, integrated strain over a relatively long gage length is measured. Since the gage length can range from centimeters to meters, the strain variation of a structural member can be considered by using the long gage sensors [14–20]. On the other hand, due to the gage length, the maximum strain in a structure cannot be measured directly from the long gage sensors. Even though FBG sensors can only measure local strains, FBG sensors would be suitable for sensing of maximum strains with a minimum number of sensors thanks to multiplexing technology [21,22]. Due to the advantages of good linearity, low cost, and the multiplexing capability, FBG sensors are the most commonly used type of strain sensor in the field monitoring of buildings and infrastructures [22].

Therefore, in this paper, a strain-based load identification model for beam structures subjected to multiple loads is presented. Using the strains measured by multiplexed FBG strain sensors, the contribution of each load on the measured strains is defined to identify the loads acting on a beams structure. The number of sensors for the load identification model is set to the same as the number of load conditions acting on the beam structure. Firstly, a numerical simulation is conducted to verify the model. Then, the load identification model is applied to monitoring of applied loads on a 4 m-long steel beam subjected to two concentrated loads. In the experiment, for comparison of the estimated loads based on the model and the applied loads from a hydraulic jack, seven FBG sensors and nine electrical strain gages (ESGs) were installed on the surface of the flange.

## 2. Strain Sensing Model for Multi-Loadings

The strain sensing model is presented to define the total strain measured at a specific point in a beam structure. The total strain can be found by superimposing the strains due to loadings acting separately. Instead of measuring the strain at a specific point where sensor is installed, to derive general form of strain sensing model, the deformed shape caused by the multiple loadings is needed to be defined by superimposing the distribution shape of strains along the length of a beam for each loading separately. Using the deformed shape of a beam structure subjected to multi-loadings, the total strain at an arbitrary point in a beam structure can be defined.

### 2.1. Distribution of Strains Caused by Single Load

**Figure 1.**
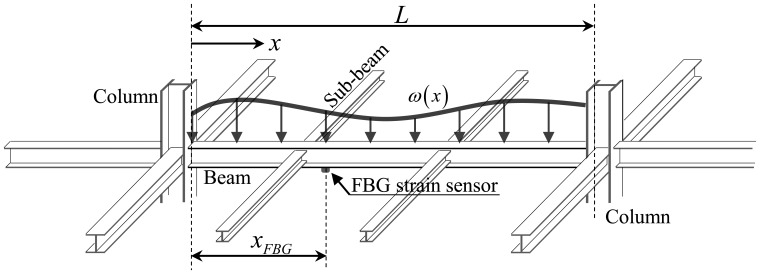
Beam structure subjected to a distributed load and point loads from sub-beams.

Based on general concepts in engineering mechanics, as shown in Figure 1, the longitudinal strain *ε*(*x*) of the beam subjected to a uniformly distributed load of *ω* can be expressed as a function of the distance *x* from the left-hand support:

(1) $$\varepsilon (x)=\frac{M(x)}{EZ}$$

where *M*(*x*) is the bending moment, *E* is the modulus of elasticity, and *Z* is the elastic section modulus. For a simply supported beam with FBG sensor installed at the length of *x_FBG_*, the strain measured from the FBG strain sensor, *ε_FBG_*, is expressed as:

(2) $$\varepsilon_{\text{FBG}}=\frac{1}{EZ}\left(\frac{\omega L}{2}x_{\text{FBG}}-\frac{\omega }{2}{x_{\text{FBG}}}^{2}\right)$$

where *L* is the length of the beam. From Equation (2), the intensity of the distributed load *ω* can be given by:

(3) $$\omega =\frac{2EZ\varepsilon_{\text{FBG}}}{x_{\text{FBG}}(L-x_{\text{FBG}})}$$

Then, the general form for the longitudinal strain *ε*(*x*) at an arbitrary point *x* can be defined by:

(4) $$\varepsilon (x)=\varepsilon_{\text{FBG}}\frac{x(L-x)}{x_{\text{FBG}}(L-x_{\text{FBG}})}=\varepsilon_{\text{FBG}}\;\phi (x)$$

where *ϕ*(*x*) is defined as a shape function for the distribution of strains along the length of the beam. The strain-shape function *ϕ*(*x*) for the distribution of strains in Equation (4) depends on the load and support conditions for a beam structure. Then, in this paper, the general form for *ε*(*x*) can be defined by the measured strain from a FBG sensor at an arbitrary location of *x_FBG_* multiplied by the shape function *ϕ*(*x*) for the distribution of strains. It is notable that the dependency on the selection of locations of FBG sensors installed at the beam structure is avoided by using the relationship given in Equation (4).

### 2.2. Distribution of Total Strains Caused by Multiple Loads

**Figure 2.**
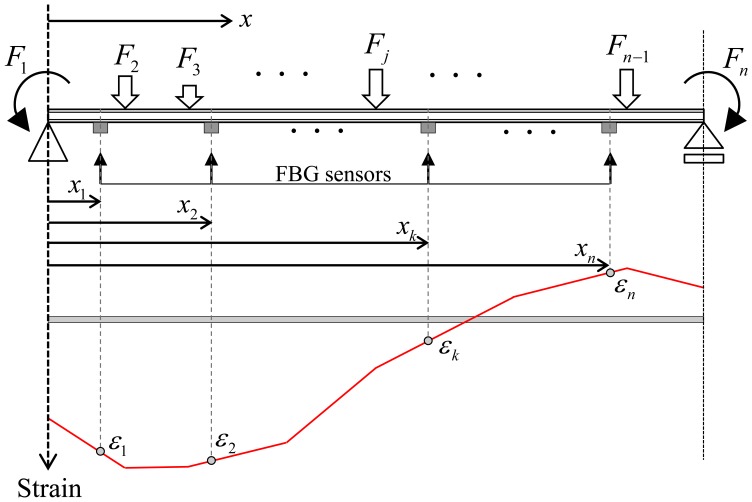
Superposition of strain distributions caused by multi-loadings.

On the basis of principle of superposition, as shown in Figure 2, total strain *ε_t_*(*x*) of the beam subjected to multiple loads at the distance *x* from the left-hand support can be expressed as the sum of the strains due to *n* different loads, *F_j_* (*j* = 1 to *n*), acting separately:

(5) $$\varepsilon_{t}(x)=\sum_{j=1}^{n}\lambda_{j}^{k}\phi_{j}^{k}(x)$$

where $\phi_{j}^{k}(x)$ is the shape of the distribution of strains caused by *j*th load and $\lambda_{j}^{k}$ is the participation factor for the *j*th strain-shape function $\phi_{j}^{k}(x)$ defined by contribution of the *j*th strain-shape function to the total strain *ε_t_*(*x*). For the superposition of strains due to *n* different loads, the number of sensors required in the load identification model is the same as the number of loads. However, as usual it is difficult not to lose strain data measured from sensors for various unexpected reasons such as abnormal installation of sensors, noise, and communication errors in sensor networks. For this reason, more than *n* sensors must be installed, even though the number of sensors used for the superposition is *n*. Among a total *n* FBG sensors installed at arbitrary locations, as shown in Figure 2, the superscript *k* in Equation (5) is the FBG sensor number selected for calculation of the strain-shape functions and the participation factors. Since strain-shape functions depend on not only the type of load but also the location of the FBG sensors *x_k_* in Figure 2, the total strain in Equation (5) can be determined in accordance with the selection of the FBG strain sensor. However, the value of total strain in Equation (5) does not vary with the selection of the FBG sensor. From Equation (5), the total strain at the *j*th FBG sensor installed at the distance *x_j_* (*j* = 1 to *n*) can be given by:

(6) $$\varepsilon_{j}=\sum_{i=1}^{n}\lambda_{i}^{k}\phi_{i}^{k}(x_{j})$$

Using measured strains from *n* FBG sensors, Equation (6) can be expanded to a linear system of equations in Equation (7):

(7) $$\left\{\begin{array}{c} \varepsilon_{1}\\ \varepsilon_{2}\\ \vdots \\ \varepsilon_{j}\\ \vdots \\ \varepsilon_{n} \end{array}\right\}=\left[\begin{array}{cccccc} \phi_{11}^{k} & \phi_{12}^{k} & \cdots & \phi_{1j}^{k} & \cdots & \phi_{1n}^{k}\\ \phi_{21}^{k} & \phi_{22}^{k} & \cdots & \phi_{2j}^{k} & \cdots & \phi_{2n}^{k}\\ \vdots & \vdots & \ddots & \vdots & \ddots & \vdots \\ \phi_{j1}^{k} & \phi_{j2}^{k} & \cdots & \phi_{jj}^{k} & \cdots & \phi_{jn}^{k}\\ \vdots & \vdots & \ddots & \vdots & \ddots & \vdots \\ \phi_{n1}^{k} & \phi_{n2}^{k} & \cdots & \phi_{nj}^{k} & \cdots & \phi_{nn}^{k} \end{array}\right]\left\{\begin{array}{c} \lambda_{1}^{k}\\ \lambda_{2}^{k}\\ \vdots \\ \lambda_{j}^{k}\\ \vdots \\ \lambda_{n}^{k} \end{array}\right\}$$

where $\phi_{ij}^{k}=\phi_{j}^{k}(x_{i})$ is the value of *j*th strain-shape function at the position of *x_i_*. Equation (7) can also be expressed by:

(8) $$\left\{\varepsilon_{\text{FBG}}\right\}=\left[\phi^{k}\right]\left\{\lambda^{k}\right\}$$

where {*ε_FBG_*} is a column vector of measured strains, [*ϕ^k^*] is a square matrix of $\phi_{ij}^{k}$, and {*λ^k^*} is a column vector of the participation factors. By solving Equation (8), participation factors, {*λ^k^*}, are determined by:

(9) $$\left\{\lambda^{k}\right\}={\left[\phi^{k}\right]}^{-1}\left\{\varepsilon_{\text{FBG}}\right\}$$

Then, the total strain *ε_t_*(*x*) of the beam subjected to multiple loads at a distance *x* can be determined by substituting the participation factors in Equation (9) into Equation (5).

## 3. Load Identification Model

In this paper, the loads acting on a beam structure are identified by the strains measured by FBG strain sensors. As given in Equation (8), the measured strain is defined by the sum of $\lambda_{j}^{k}\phi_{j}^{k}(x)$. The *j*th strain-shape function $\phi_{j}^{k}(x)$ is dependent on a type of load. Using a virtual unit load *f_j_* that is the same type as the actual load *F_j_* that acts on the beam in Figure 2, $\phi_{j}^{k}(x)$ in Equation (8), it can be expressed as:

(10) $$\phi_{j}^{k}(x)=a_{j}^{k}\bar{M_{j}}(x)$$

where, $a_{j}^{k}$ is a scale factor and $\bar{M_{J}}$ is an analytical function of moment induced by the virtual unit load *f_j_*. Then, based on the general concept of engineering mechanics, the strain distribution caused by the load *F_j_*, $\lambda_{j}^{k}\phi_{j}^{k}(x)$, is given by:

(11) $$\lambda_{j}^{k}\phi_{j}^{k}(x)=\frac{m_{j}}{EZ}\bar{M_{j}}(x)$$

where *m_j_* is an intensity of load *F_j_*. *E* and *Z* are elastic modulus and section modulus of the beam structure, respectively. Then, the intensity or magnitude of the load *F_j_*, *m_j_*, is found by:

(12) $$m_{j}=EZa_{j}^{k}\lambda_{j}^{k}$$

### 3.1. Simulation with a Simply Supported Beam

**Figure 3.**
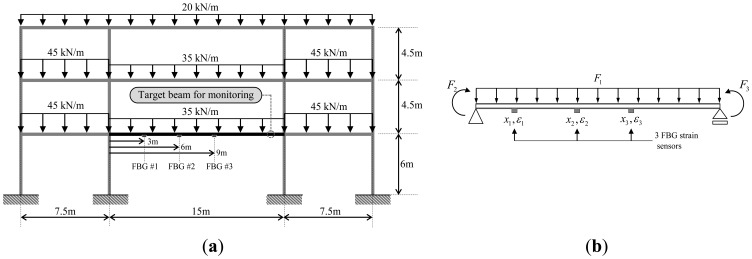
Numerical example for simulation. (**a**) Target two-dimensional steel beam frame. (**b**) Equivalent structural model of the beam.

The beam member in a building frame in Figure 3a subjected to a uniformly distributed load can be modeled as the simply supported beam subjected to three different loads in Figure 3b: the distributed load *F_1_*, the moment *F_2_* acting at the left end, and the moment *F_3_* acting at the right end of the beam. For measurement of strains, three FBG sensors are attached at the three different locations in Figure 3b.

#### 3.1.1. Strain-Shape Functions

Total strain distribution of the beams in Figure 3b is found by the sum of the strains caused by the three different loads acting separately. If the 1*st* FBG sensor is selected for calculation of the strain-shape functions and the participation factors, strain-shape functions for the distributed loading *F*_1_ in Equation (4) are given by:

(13) $$\phi_{1}^{1}(x)=\frac{x(L-x)}{x_{1}(L-x_{1})}$$

In a similar manner, strain-shape functions for the end moments *F*_2_ and *F*_3_ are given by:

(14) $$\phi_{2}^{1}(x)=\frac{L-x}{L-x_{1}}$$

(15) $$\phi_{3}^{1}(x)=\frac{x}{x_{1}}$$

Then, the participation factors for the three loads $\lambda_{1}^{1}$, $\lambda_{2}^{1}$, and $\lambda_{3}^{1}$ are found from Equation (9).

#### 3.1.2. Load Intensity

Using the scale factors $a_{j=1}^{k}$(*k* = 1 to 3)for strain-shape functions in Equations (13–15), the intensities of loads *F*_1_, *F*_2_, and *F*_3_ are directly given by:

(16) $$m_{1}=EZ\lambda_{1}^{1}\frac{2}{x_{1}(L-x_{1})}$$

(17) $$m_{2}=EZ\lambda_{2}^{1}\frac{L}{L-x_{1}}$$

(18) $$m_{3}=EZ\lambda_{3}^{1}\frac{L}{x_{1}}$$

For the two-dimensional steel frame structure subjected to a uniformly distributed load shown in Figure 3a, the sections for all beams and columns are commercially available rolled shapes of H-708 × 302 × 15 × 28 and H-400 × 400 × 13 × 21, respectively. The elastic modulus and section modulus of the beam are 2.05 × 10^8^ kN/m^2^ and 6.70 × 10^−3^ m^3^, respectively. Three FBG sensors were assumed to be attached at 3, 6, and 9 m from left-hand end of the target beam. From the strain distribution obtained from the structural analysis, the strains for FBG #1, #2, and #3 are 23.2, 266.6, and 266.6 με, respectively.

As shown in Figure 3, the target beam is transformed to a simply supported beam subjected to a uniformly distributed load *F*_1_ and two end moments *F*_2_ and *F*_3_. Then, for the beam with span length of 15 m and the sensor locations of 3, 6, and 9 m, the participation factors in Equation (9) are given by:

(19) $$\left\{\begin{array}{c} \lambda_{1}^{1}\\ \lambda_{2}^{1}\\ \lambda_{3}^{1} \end{array}\right\}={\left[\begin{array}{ccc} 1.0000 & 1.0000 & 1.0000\\ 1.5000 & 0.7500 & 2.0000\\ 1.5000 & 0.5000 & 3.0000 \end{array}\right]}^{-1}\left\{\begin{array}{c} 23.2\\ 266.6\\ 266.6 \end{array}\right\}=\left\{\begin{array}{c} 486.6\\ -370.7\\ -92.7 \end{array}\right\}$$

Then, using the length of the beam *L* = 15 m and the location of the FBG sensor *x*_1_ = 3 *m*, the intensities of loads *F*_1_, *F*_2_, and *F*_3_ in Equations (16–18) are calculated as 37.1 kN/m, −636.0 kN·m, and 636.0 kN·m, respectively. The load intensity of 37.1 kN/m includes the live load of 35 kN/m in Figure 3 and the weight of the steel beam. The weight density and the cross-sectional area of the steel beam are 77.0 kN/m^3^ and 27.4 × 10^3^ mm^2^, respectively. The values for the loads are identical with the results from structural analysis with only a difference of 0.03 kN·m in *F*_2_ and *F*_3_ due to the numerical error.

## 4. Experimental Test

### 4.1. Test Setup

**Figure 4.**
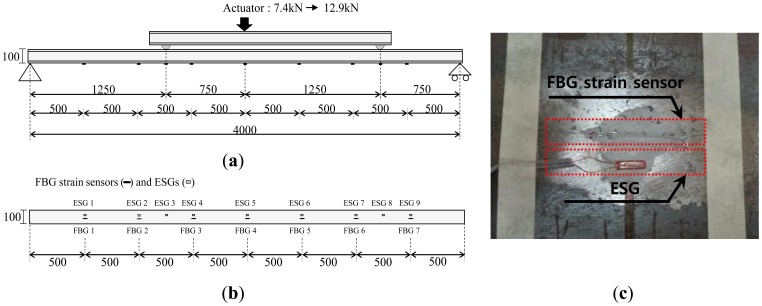
Experimental setup. (**a**) Side view. (**b**) Bottom view. (**c**) Installation of fiber Bragg grating (FBG) sensors and electrical strain gages (ESGs).

**Figure 5.**
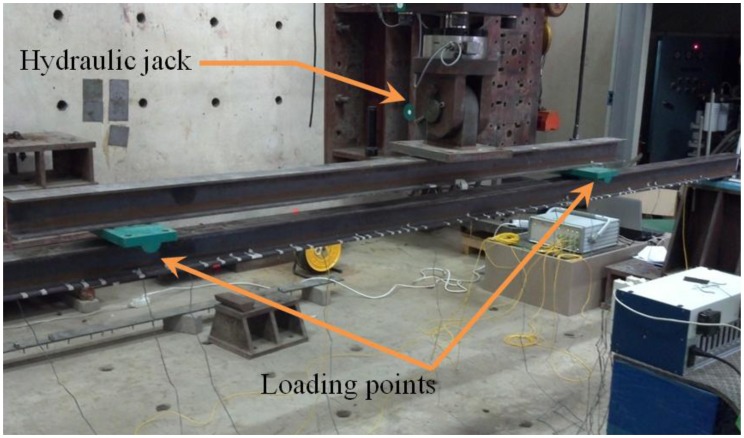
Loading by hydraulic jack and loading points.

To verify the performance of measurement model, a bending test of the simply supported steel beam subjected to two concentrated loads was conducted. Figure 4 shows the schematic diagram of the bending test. The beam model consists of a simply supported H-100 × 100 × 6 × 8 section having a length of 4 m. A concentrated load was applied on upper steel beam by hydraulic jack as shown in Figure 4a and Figure 5. The load was increased in two steps: 7.4 kN and 12.9 kN. The locations of two concentrated loads from the left end of the beam in the experimental setup in Figure 4 are 1.25 m and 3.25 m. Measurements during static testing were performed with seven FBG sensors and nine ESGs. As shown in Figure 4b,c, FBG strain sensors and ESGs were attached on the surface of the bottom flange.

### 4.2. Results

**Figure 6.**
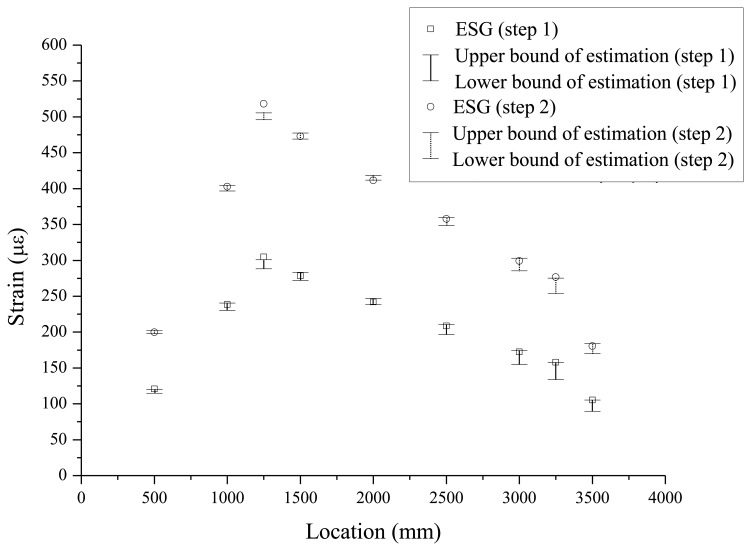
Comparison between estimated and measured strains.

When the load is applied by means of a hydraulic jack, the beam deflects downward and tensile strains occur at the outer surface of the bottom flange. For each load step, to confirm the quality of measurements during the test, the measured strains from seven FBG sensors and nine ESGs are compared in Figure 6. The maximum differences between the two measurements were found be less than 1.5% for each load step.

**Figure 7.**
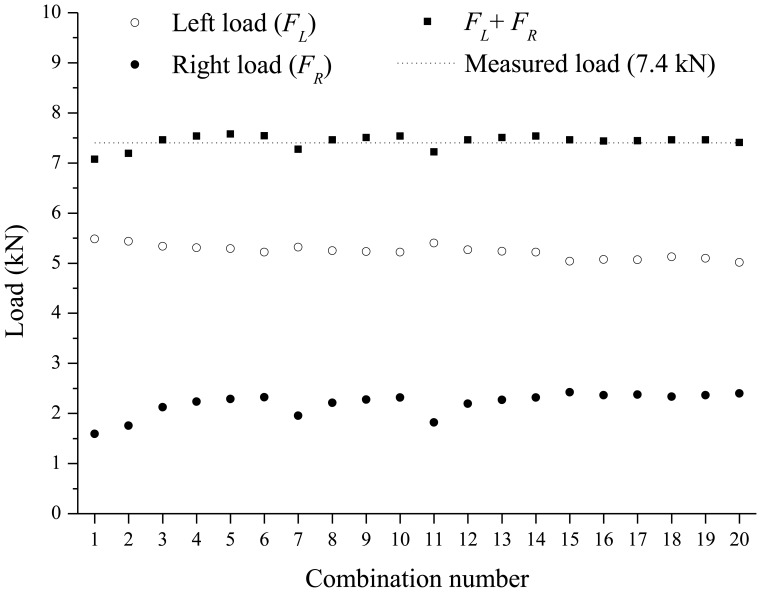
Identified loads according to 20 combinations (loading step 1).

**Figure 8.**
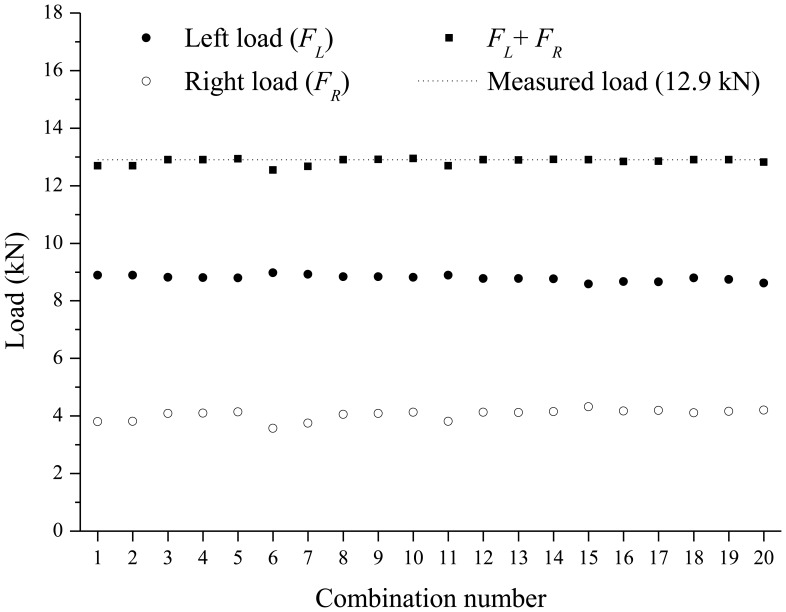
Identified loads according to 20 combinations (loading step 2).

In this experimentation, two FBG sensors are required to identify the two concentrated loads using the load identification model in Equations (9) and (12). Then, there are 21 possible combinations when choosing two FBG sensors from among seven FBG sensors without repetition. Among these 21 combinations, the combination of the first and second FBG sensors in Figure 4a is not valid for the measurement since the matrix [*ϕ^k^*] in Equation (8) based on the combination is singular. For each loading step, using 20 combinations for the selection of two FBG sensors, the estimated left and right concentrated loads, the sum of the two estimated loads, and measured load applied by the hydraulic jack are compared in Figure 7 and Figure 8. Based on estimated loads from the 20 combinations, the averages of the intensities of the left and the right loads for the first loading step are 5.2308 kN and 2.1964 kN, respectively. The coefficient of variation (COV)s of the two loads are calculated as 0.0252 and 0.1059, respectively. For the second loading step, the averages of the intensities of the left and the right loads are 8.7917 kN and 4.0433 kN, respectively. The coefficient of variation (COV)s of the two loads are calculated as 0.0115 and 0.0466, respectively.

Comparing with the applied loads of 7.4 kN and 12.9 kN for the two loading steps, the summations of left and right loads are found to be very close to the ones applied from the hydraulic jack; average values of the sum of the two estimated loads are 7.427 kN with COV of 0.0180 for the first loading step and 12.84 kN with COV of 0.0087 for the second loading step.

## 5. Conclusions

In this paper, a strain-based load identification model for beam structures subjected to multiple loads is presented. The identification model is derived by defining the contribution of each load to the strains measured by strain sensors and the strain-shape functions for each load. In this paper, to avoid the dependency on the selection of locations of fiber Bragg grating (FBG) sensors installed at the beam structure, the deformed shape caused by the multiple loads is defined by superimposing the distribution shape of strains along the length of a beam for each load separately. The type and location of the load applied to a beam structure are necessary to define the distribution shapes. Using the deformed shape of a beam structure subjected to multiple loads, the total strain at an arbitrary point in a beam structure can be defined. Based on the results from both the numerical simulation and an experimental test, it is found that results indicate a good agreement between estimated loads based on the model and the loads applied by a hydraulic jack.
